# Supplementary material for: A Machine Learning-Based Diagnostic Model for Crohn’s Disease and Ulcerative Colitis Utilizing Fecal Microbiome Analysis
Source: Microorganisms. 2023 Dec 24;12(1):36. doi: 10.3390/microorganisms12010036 (PMC10820568; doi:10.3390/microorganisms12010036)
Supplement: Supplementary file 1 [file microorganisms-12-00036-s001.zip › microorganisms-2719421-supplementary.pdf]

SUPPLEMENTARY MATERIALS

Supplementary Table S1. The top 10 genera chosen with high frequency in the sPLS-DA models for distinguishing between IBD and HC

**IBD**	**Frequency**	**HC**	**Frequency**
Actinomyces	*100*	*Prevotella*	*97*
Streptococcus	*100*	*Oscillospiraceae UCG-005*	*97*
Rothia	*99*	*Prevotella_9*	*95*
Lactobacillus	*96*	*Alistipes*	*94*
Levilactobacillus	*96*	*Parabacteroides*	*93*
Leuconostoc	*93*	*Paraprevotella*	*93*
Carnobacterium	*93*	*Tuzzerella*	*93*
Gemella	*84*	*Lachnospiraceae UCG-010*	*92*
Atopobium	*83*	*Butyricimonas*	*91*
Mycobacterium	*83*	*Odoribacter*	*91*

Supplementary Table S2. The top 10 genera chosen with high frequency in the sPLS-DA models for distinguishing between CD and UC

**CD**	**Frequency**	**UC**	**Frequency**
[Eubacterium] xylanophilum	*94*	*Sediminibacterium*	*100*
Anaerosporobacter	*94*	*Simkaniaceae_uncultured*	*94*
[Ruminococcus] torques group	*94*	*Undibacterium*	*94*
Lachnospiraceae FCS020 group	*94*	*Lautropia*	*94*
Oscillospiraceae NK4A214	*88*	*Rhodoferax*	*94*
Dorea	*88*	*Noviherbaspirillum*	*94*
Herbinix	*86*	*Geothrix*	*94*
Coprococcus	*80*	*Lacunisphaera*	*94*
Lachnospiraceae_uncultured	*77*	*Magnetospirillum*	*92*
Anaerostipes	*75*	*Filifactor*	*91*
